# The Dental College Standard

**Published:** 1901-07

**Authors:** Chas. C. Chittenden

**Affiliations:** Madison, Wis.


### THE DENTAL COLLEGE STANDARD.[1]

BY CHAS. C. CHITTENDEN, D.D.S., MADISON, WIS.

(*a*) Is it what it should be?

(*b*) If not, what improvements should be made?

(*c*) How may the requirements be improved?

In order to intelligently consider the subject in hand it will be well to first define if possible what is the dental college educational standard as at present accepted in this country?

Much agitation and discussion of the subject has for the past two or three years occupied the attention of the journals and the various dental societies, from the national bodies down to the smaller local organizations, as to what the standard should be. The fact also is that until 1899 there never was any established standard in this country, each school being a law unto itself as to whom it should matriculate and graduate. The national examiners sought for years to induce the National Faculties Association to establish a minimum standard as a basis from which to go forward, but it was not accomplished until an open rupture brought

---

[1] Abstract of paper read before the American Medical Association, Section on Stomatology, St. Paul, Minn., June 4, 5, 6, and 7, 1901.

the two national bodies together at Niagara Falls in 1899 in a conference, which resulted in the following code of rules being placed on the records of the Faculties Association:

"The minimum preliminary educational requirement of colleges of this Association, for the session of 1899-1900, shall be a certificate of entrance into the second year of a high school, or its equivalent, the preliminary examination to be placed in the hands of any State or county superintendent of public instruction."

Amended Rule VIII., National Association Dental Examiners, Sec. 2, is as follows:

"The following preliminary examination shall be required of students seeking admission to colleges recommended by this Association: The minimum preliminary educational requirements of colleges of this Association for the session of 1900-1901 shall be certificate of entrance in the second year of a high school, or its equivalent, the preliminary examination to be placed in the hands of the State Superintendent of Public Instruction, as adopted by the State Board of Missouri."

Also, "The minimum preliminary educational requirement of colleges of this Association, beginning with the session of the year 1902-1903, shall be a certificate of entrance into the third year of a high school, or its equivalent, the preliminary examination to be placed in the hands of the State Superintendent of Public Instruction.

"Nothing in this rule shall be construed to interfere with colleges of this Association that are able to maintain a higher standard of preliminary education."

The above represents the exact situation at this time,—to wit, present standard, one year of high school; promised standard for 1902, two years of high school, with the agreement between the two bodies that all schools belonging to the National Association of Dental Faculties and living up to its standards and requirements as laid down shall be accepted as reputable by the examiners and recommended to the various State boards for recognition. The regents of Michigan, under the advice of the venerable dean, Jonathan Taft (to whom be all honor), took the initiative in setting the pace, and required high school graduation for 1900. The regents of New York will in 1902 culminate a progressive advance in requirements to full high school standard. At the

World's Dental Congress in Paris last summer high school graduation was made the international standard for entering dental colleges. So it would seem quite certain that, viewed at large, high school graduation is accepted as our *ultima Thule.*

The National Association of Dental Faculties is a body composed of the membership of fifty-one dental schools, all of which accept and agree to conform, in the conduct of their schools, to the regulations and rules formulated by that body. However much these schools may vary in their equipment, faculty, and general effectiveness as educational institutions, yet, as long as they retain their membership in that body, all, by special agreement with the examiners, command the same privileges and recognition subject to the various dental laws in nearly all the States, membership in that body being now accepted by all State boards as the criterion of reputability before the law. This Association, backed at all times by the National Association of Dental Examiners in its every forward move, has in the eighteen years of its existence wrought wonders in the development and unification of *curricula,* the evolution of dental technics, lengthening course of study and special training, and in a hundred ways making the advantages offered for obtaining a complete dental education and equipment almost ideal; so that the student who comes to the dental college to-day prepared and fitted to assimilate and appropriate what may be his own for the mere taking, has no excuse for leaving school unprepared to enter successfully on the practice of dentistry.

The code of rules and standing resolutions formulated by this Association for the government of its members in the conduct of their schools is a monument of sense, wisdom, and ethics which would redound to the credit of any deliberative body of men having the interest of their calling at heart, and yet, strange to say, never, through all the years of its existence, has it seemed to occur to it to effectively inaugurate and establish any given educational preparation as a minimum standard requirement for entering the dental college course, until the one-year-of-high-school rule above quoted was adopted, and that only after the demonstration of much friction on the subject between the Examiners and Faculties. The query arises naturally why this important subject was so long ignored. But one logical answer suggests itself,—namely, the different schools, or, at least, a majority of them, preferred to retain in their own hands the decision, without dictation from any source, as to the fitness of an applicant for matriculation. This power of discretion was safely lodged in schools that were State institutions with equipment and financial resource sufficient to meet all current expenses and salaries, regardless of the number of students matriculating, and those with assured attendance large enough to leave out any temptation to admit applicants for financial reasons only. But only a small proportion of the schools have been thus situated. Indeed, most of them must have a given number of students to keep them from bankruptcy, and so, at least, there was a door of temptation open for the acceptance of credentials that ought to have been better than they often were. In fact, at Niagara, in 1899, the chief argument used by college representatives against the fixing of a two-years high school minimum requirement was that it would prove the financial ruin of one-half the schools of this country. But what is the fact? Since the first bar was put up the attendance in dental schools has not only not diminished, but has increased,—at least, in all the better-equipped schools. The experience of the Medical Department of Johns Hopkins is a great object-lesson in this regard. Some years ago, because of the increasing size of its classes, and in the hope of restricting them, the entrance requirement was raised first to high school graduation and then to the possession of an academic degree, all of which only served to increase the number of applicants for matriculation, thus proving a well-known axiom, that the more highly one holds his wares the more eagerly are they sought.

There is probably no doubt that if the higher standard could be successfully inaugurated and established, it would work a very marked improvement in the quality of student material entering our schools, a consummation, from the stand-point of us all, most "devoutly to be wished."

Accepting, from what appears to be the inevitable tendency of events, the high school graduation standard as a condition to be desired, the question at once presented, How can it be inaugurated without working injury to the college interest? The answer is that the colleges, by joining forces with the boards whose duty it is to enforce the dental laws, can at once establish and maintain, without friction or disturbance, to the interest of all concerned, an immediate advance of matriculation standards to high school graduation. This as a general proposition will stand.

However doubtful may be the comparative power and strength, legally and morally, of the faculties and examiners when at variance with each other, there is nevertheless no limit to their power and ability to maintain any given standard of educational requirement within reason, as a basis of accepted reputability before the public, made and provided these two representative forces agree to work honestly together to that end. This fact has been lost sight of too long by the colleges. No matter if the appointment of examiners is dominated by political pulls, or if many of their men are incompetent, pig-headed, and far from fit representatives of our ideals (as has been often charged), still it must be always remembered that these men represent and are intrusted with the administration of the law, and the law always dignifies its chosen administrators. No matter if the many State laws bear evidence of weakness and inefficiency, and are apparently at great variance with each other in their provisions and standards, if scrutinized carefully they will all be found to demand that colleges shall be reputable. This one feature of uniformity in ethics makes it possible for the teachers and examiners, backed by the moral support of the profession at large, to establish and maintain in every State of the Union any educational as well as ethical standards on which they are agreed to be of one mind.

While the Faculties Association's rules give the individual college absolute control over the behavior, standing, advancement, and transfer of the entered students,—thus making it impossible for one of them who has fallen short of passing muster in one college to be admitted to another school without the full consent of his Alma Mater,—while, I say, all this obtains after a student has matriculated and been given standing, there is at present nothing to absolutely prevent a man whose credentials have been refused by one school bobbing up serenely before the dean of another school, and having standard given him on the same credentials. This commercial and competitive temptation, which is the chief obstacle in the way of successfully establishing a uniform advanced standard requirement, can easily and only be overcome by such wise and careful legislation on the part of the Faculties' Association as has distinguished it in handling many other no less delicate questions. In the many discussions of this subject of preliminary education the argument has been made, and stoutly supported by the facts, that a large number of the most eminently successful of our profession are men who had practically no educational opportunities before embarking on a professional career, and, therefore, that to set a fixed and arbitrary standard would in effect often bar out those who might become, with opportunity, shining lights. Of course, all will admit that such men would be much better equipped with proper educational preparation, and, indeed, for such very good reason every fixed standard that has been agreed upon or that is contemplated for the future is modified by the words, "or its equivalent," to give the aspiring student his chance, no matter if his educational opportunities have been curtailed by environment.

How shall a fair, honest judgment as to what constitutes the equivalent of any given standard be secured, and who shall pass such judgment? On this question being properly answered hinges the successful establishment of any prerequisite standard. All will admit that, however well equipped a man may be as instructor in any branch of dental education, yet, by that very fact, he is sure to be at least rusty and out of practice on public school examinations; and certainly the college teacher will admit that the politically appointed examiner is likely to be fully as unfitted as himself.

Mind, the new Rule VIII., jointly adopted at Niagara in 1899, provides thus: "The preliminary examination to be placed in the hands of the State Superintendent of Public Instruction, as adopted by the State Board of Missouri." Manifestly—since it is not to be presumed that the dental profession has the power to prescribe special duties for any State officer—the intention and expectation was that the State Superintendent of Public Instruction of each State having dental schools should be requested to select and appoint some person specially fitted, in his judgment, to pass upon credentials and properly conduct the preliminary examinations of intending dental matriculants in the colleges of that State. Of course, the State Superintendent is a "political creation," but his special appointee for this particular work may practically be satisfactorily agreed upon between the college and State board. Thus would the college dean be relieved from the ever-perplexing responsibility of passing upon all sorts of so-called credentials, such as every dean has presented to him, credentials many of them so plausible as to easily pass muster if only casually scanned, and which the dean feels certain, should he refuse them, would be cheerfully accepted, along with the student and the fee, by another dean somewhere.

Eliminate all this. Make it absolutely impossible that a matriculant can be given standing in any college on credentials until these credentials shall have been passed upon, first, by the accredited representative of the State superintendent, and then finally accepted or rejected by the joint conference committee of college and board. Make their decision, in the interstate and intercollegiate sense, final, and add, if you choose, a reasonable probationary clause providing the applicant a way to make up his shortcomings without abandoning the study of dentistry. In other words, make the general rule jointly accepted at Niagara practically effective and universal by the two national bodies formulating specific rules for its application in every State, territory, and district where a dental school exists. This plan has been successfully operated in more than one State, and if it, or some other one equally practicable, could be inaugurated and applied to all schools alike, one of the chief temptations to commercialism and competition rather than emulation would be eliminated, and each school would stand or fall according as its intrinsic merits command recognition.

